# NRFL-1, the *C*. *elegans* NHERF Orthologue, Interacts with Amino Acid Transporter 6 (AAT-6) for Age-Dependent Maintenance of AAT-6 on the Membrane

**DOI:** 10.1371/journal.pone.0043050

**Published:** 2012-08-15

**Authors:** Kohei Hagiwara, Shushi Nagamori, Yasuhiro M. Umemura, Ryuichi Ohgaki, Hidekazu Tanaka, Daisuke Murata, Saya Nakagomi, Kazuko H. Nomura, Eriko Kage-Nakadai, Shohei Mitani, Kazuya Nomura, Yoshikatsu Kanai

**Affiliations:** 1 Division of Bio-system Pharmacology, Department of Pharmacology, Graduate School of Medicine, Osaka University, Osaka, Japan; 2 School of Medicine, Osaka University, Osaka, Japan; 3 Graduate School of Systems Life Sciences, Kyushu University, Fukuoka, Japan; 4 Core Research for Evolutional Science and Technology (CREST), Japan Science and Technology Agency (JST), Saitama, Japan; 5 Department of Biological Sciences, Faculty of Sciences, Kyushu University, Fukuoka, Japan; 6 Department of Physiology, Tokyo Women’s Medical University School of Medicine, Tokyo, Japan; Brown University, United States of America

## Abstract

The NHERF (Na^+^/H^+^ exchanger regulatory factor) family has been proposed to play a key role in regulating transmembrane protein localization and retention at the plasma membrane. Due to the high homology between the family members, potential functional compensations have been a concern in sorting out the function of individual NHERF numbers. Here, we studied *C*. *elegans* NRFL-1 (C01F6.6) (nherf-like protein 1), the sole *C*. *elegans* orthologue of the NHERF family, which makes worm a model with low genetic redundancy of NHERF homologues. Integrating bioinformatic knowledge of *C*. *elegans* proteins into yeast two-hybrid scheme, we identified NRFL-1 as an interactor of AAT-6, a member of the *C*. *elegans* AAT (amino acid transporter) family. A combination of GST pull-down assay, localization study, and co-immunoprecipitation confirmed the binding and characterized the PDZ interaction. AAT-6 localizes to the luminal membrane even in the absence of NRFL-1 when the worm is up to four-day old. A fluorescence recovery after photobleaching (FRAP) analysis suggested that NRFL-1 immobilizes AAT-6 at the luminal membrane. When the *nrfl-1* deficient worm is six-day or older, in contrast, the membranous localization of AAT-6 is not observed, whereas AAT-6 tightly localizes to the membrane in worms with NRFL-1. Sorting out the *in vivo* functions of the *C*. *elegans* NHERF protein, we found that NRFL-1, a PDZ-interactor of AAT-6, is responsible for the immobilization and the age-dependent maintenance of AAT-6 on the intestinal luminal membrane.

## Introduction

Proper localization and maintenance of transmembrane proteins in the plasma membrane are essential for appropriate cellular function. Transmembrane proteins often participate in a functional macromolecular complex with other transmembrane or membrane-associated proteins. One of the mechanisms regulating such protein localization and complex formation is via scaffold proteins that possess single or multiple protein-protein interaction domains and serve as scaffold to assemble and/or stabilize proteins. Among the most commonly encountered protein-protein interaction modules is the PDZ (Post-Synaptic Density-95/Discs Large/Zonula Occludens-1) domain. Typically, PDZ domains achieve selective bindings by recognizing the carboxyl terminal four to seven residues of target proteins [1]–[3].

The mammalian NHERF (Na^+^/H^+^ exchanger regulatory factor) family, which consists of NHERF1, NHERF2, PDZK1 and IKEPP, is a family of PDZ proteins. NHERF1 and NHERF2 have two PDZ domains in tandem, whereas PDZK1 and IKEPP have four tandem PDZ domains. They have overlapping tissue and subcellular distributions; the four members are found in the brush border membrane of the intestine and the renal proximal tubule [4]. The highly homologous primary structures of their PDZ domains allow them to share some of the target proteins such as CFTR (cystic fibrosis transmembrane conductance regulator) [5]–[7], NHE3 (sodium-hydrogen exchanger 3) [8]–[10] and organic solute transporters [11]–[13]. This redundancy in expression profile and interaction, consequently yielding potential functional compensations between the family members, has made it difficult to separate the *in vivo* functions of individual NHERF family proteins. Indeed, deletion of *nherf* genes in mouse associates with mild phenotypic changes; NHERF1-null male mice develop healthy but females show increased mortality or weakness [14], [15]; NHERF2 or PDZK1-deficient mice appear normal [16], [17]. Only recently, researchers have started addressing this issue by generating multiple-gene knockout animals. Broere et al. [16] and Singh et al. [18] suggested that the NHERF family members play differential, rather than compensatory, roles in CFTR regulation. This observation seems inconsistent with findings from the single-knockout studies as the knockout animals would demonstrate more noticeable phenotypes if no or little compensations take place.

To better understand the *in vivo* functions of scaffold proteins of NHERF family members, we looked at *C*. *elegans* NRFL-1 (C01F6.6) (nherf-like protein 1). Because NRFL-1 is the single worm orthologue of NHERF family, studies in *C*. *elegans* should be less susceptible to the redundancy problem that we encounter in the mammalian NHERF family. In the present study, NRFL-1 was identified as a binding partner of AAT-6 (T11F9.4) (amino acid transporter 6). AAT-6 is one of the transporters with PDZ-binding motif in the *C*. *elegans* AAT (amino acid transporter) family that consists of nine genes. This family is closely homologous to the mammalian SLC7 family of amino acid transporters [19], [20]. *C*. *elegans* is a transparent model organism amenable to genetic manipulation and live-animal imaging. Taking advantage of these properties, we examined the role of PDZ interaction in the localization of AAT-6 in the plasma membrane. Similar to NHERF-mediated interactions in polarized cell lines such as OK cells and MDCK cells [21], [22], we show that NRFL-1 scaffolds AAT-6 to be less mobile in the plasma membrane through a PDZ interaction in living worm. Besides, as an age-associated property of NHERF-related protein, NRFL-1 is found to be responsible for the retention of AAT-6 on the intestinal luminal membrane in aged but not in young worm, suggesting a protective role of NRFL-1 against the deterioration of membranous localization AAT-6 with age.

## Results

### Identification of NRFL-1 as an AAT-6 Interactor

**Figure 1:**
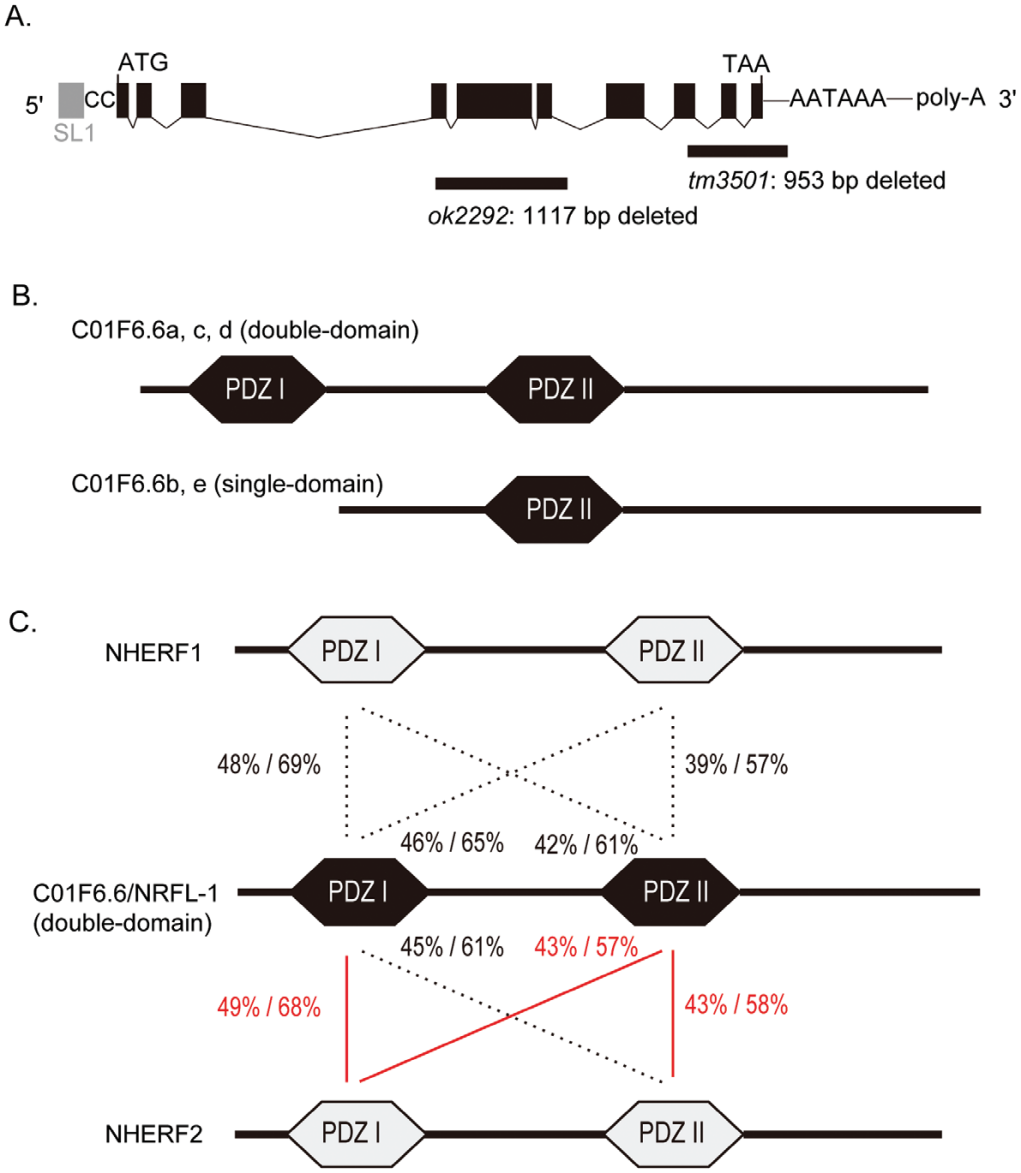
NRFL-1, the worm orthologue of NHERF family. ***A***, Schematic illustration of *nrfl-1* transcript (modified from WormBase). Black boxes, exons; grey box, spliced leader 1 (SL1); CC, two bases of cytosine constituting the 5′UTR; ATG, the start codon; TAA, the stop codon; AATAAA, a putative polyadenylation signal; poly-A, poly adenosine tail. The alleles *tm3501* and *ok2292* lack 953-basepair and 1117-basepair of the genomic DNA, respectively, indicated by the horizontal lines. ***B***, NRFL-1 protein. The *nrfl-1*gene products contain either a single or double PDZ domains with PDZ II shared by all. The variants C01F6.6a, c, and e have PDZ I and PDZ II (double-domain group). PDZ I is structurally related to PDZ II with 45% identity and 59% similarity. ***C***, Pairwise domain comparison between NRFL-1 and two human NHERF proteins: NHERF 1 and NHERF 2. PDZ domains were specified by ExPASy Prosite [67] and compared by BLAST [26]. For each pair, identity/similarity (%/%) is assigned. The red lines indicate the most remarkable pair(s) for each of NRFL-1 PDZ domains with respect to sequence identity.

The *C*-terminus of AAT-6 protein ends in threonine, arginine, and methionine (-T-R-M), which makes up a class I PDZ binding motif (-S/T-X-Ф-COOH, where X denotes any residue and Ф denotes a hydrophobic residue) [23]. In search for proteins that associate with AAT-6 via PDZ interactions, *C*. *elegans* proteins were first screened for PDZ domain by a molecular architecture research database, SMART [24]. This process found 72 PDZ domain-containing proteins. Next, the proteins were screened for intestinal expression because our preliminary expression analysis suggested that AAT-6 is expressed in the intestine. Expression data sets were provided by WormBase and NEXTDB (The Nematode Expression Data Base; nematode.lab.nig.ac.jp/). Sixteen proteins were selected as intestinal PDZ proteins (Table 1). For candidates with multiple splice variants such as C01F6.6, we sub-grouped the variants in terms of PDZ domain identity. The variants are classified into the same sub-group if the variants are identical with respect to the number and the primary structures of their PDZ domains (e.g., “single-domain” and “double-domain” groups in Fig. 1B). For each sub-group, one of the variants was tested for potential interaction. Each of the sixteen candidates (prey) was examined for potential interaction with the AAT-6 *C*-terminus tail (bait) in the yeast two-hybrid system [25]. Through the matching process, C01F6.6a appeared strongly positive and so was C01F6.6b but to a lesser degree (Table 1). C01F6.6a (NRFL-1) was, thus, subjected to further investigation.

**Table 1:** Yeast two-hybrid matching.

	PDZ protein	Gene	LEU2/GFP
1	C01F6.6a	*nrfl-1*	+/+
2	C01F6.6b	*nrfl-1*	+/−
3	C09H6.2a	*lin-10*	−/−
4	C25F6.2a	*dlg-1*	−/−
5	C25F6.2b	*dlg-1*	−/−
6	C25G4.6	C25G4.6	−/−
7	C27A2.6	C27A2.6	−/−
8	C35D10.2	C35D10.2	−/−
9	F26D11.11a	*let-413*	−/−
10	F30A10.8b	*stn-1*	−/−
11	F30F8,3	F30F8.3	−/−
12	F44D12.1	F44D12.1	−/−
13	F44D12.4	F44D12.4	−/−
14	T14G10.2a	*pfx-1*	−/−
15	T21G5.4	T21G5.4	−/−
16	T26E3.3a	*par-6*	−/−

Each of the sixteen intestinal PDZ proteins (prey) was subjected to a yeast two-hybrid assay with AAT-6 as a bait. Pairs were assessed for LEU2 and GFP reporter genes: + and −, positive and negative for LEU2 or GFP, respectively.

The *nrfl-1* gene encodes C01F6.6a in ten exons. The *nrfl-1* mRNA is trans-spliced to the spliced leader SL1 with two bases of cytosine between the spliced leader and the start codon (yk1663b04, NEXTDB). The 3′UTR contains a putative polyadenylation signal, AATAAA, 15 nucleotides upstream of the poly-A tail (yk1651h10, NEXTDB) (Fig. 1A). The gene products of *nrfl-1*contain five variants (Fig. 1B). All the variants share the *C*-terminal PDZ domain (PDZ II). Three of the variants have the *N*-terminal PDZ domain (PDZ I) is structurally related to PDZ II with 45% identity and 59% similarity.

A BLAST [26] search against human proteins using the NRFL-1 protein sequence as a query revealed the NHERF family (NHERF1, NHERF2, PDZK1, and IKEPP) with the closest homology. In the family, NHERF1 and NHERF2 appeared particularly similar to NRFL-1. Reciprocal BLAST searches against worm proteins using the NHERF family protein sequences as queries also found NRFL-1 as the most related *C*. *elegans* protein. This bidirectional homology assessment suggested that NRFL-1 is a *C*. *elegans* orthologue of the NHERF family proteins. Individual NRFL-1 PDZ domains were compared with the domains of NHERF1 and NHERF2. In terms of PDZ domain, NHERF2 appeared slightly more related to NRFL-1; for NRFL-1 PDZ I, NHERF2 PDZ I was the most similar with 49% identity (68% similarity), and for NRFL-1 PDZ II, both of the NHERF2 PDZ domains with 43% identity (57–58% similarity) (Fig. 1C). PDZK1 and IKEPP showed somewhat modest yet considerable relatedness to NRFL-1 (Table S1).

### NRFL-1 Mainly Binds AAT-6 with PDZ II Domain

**Figure 2:**
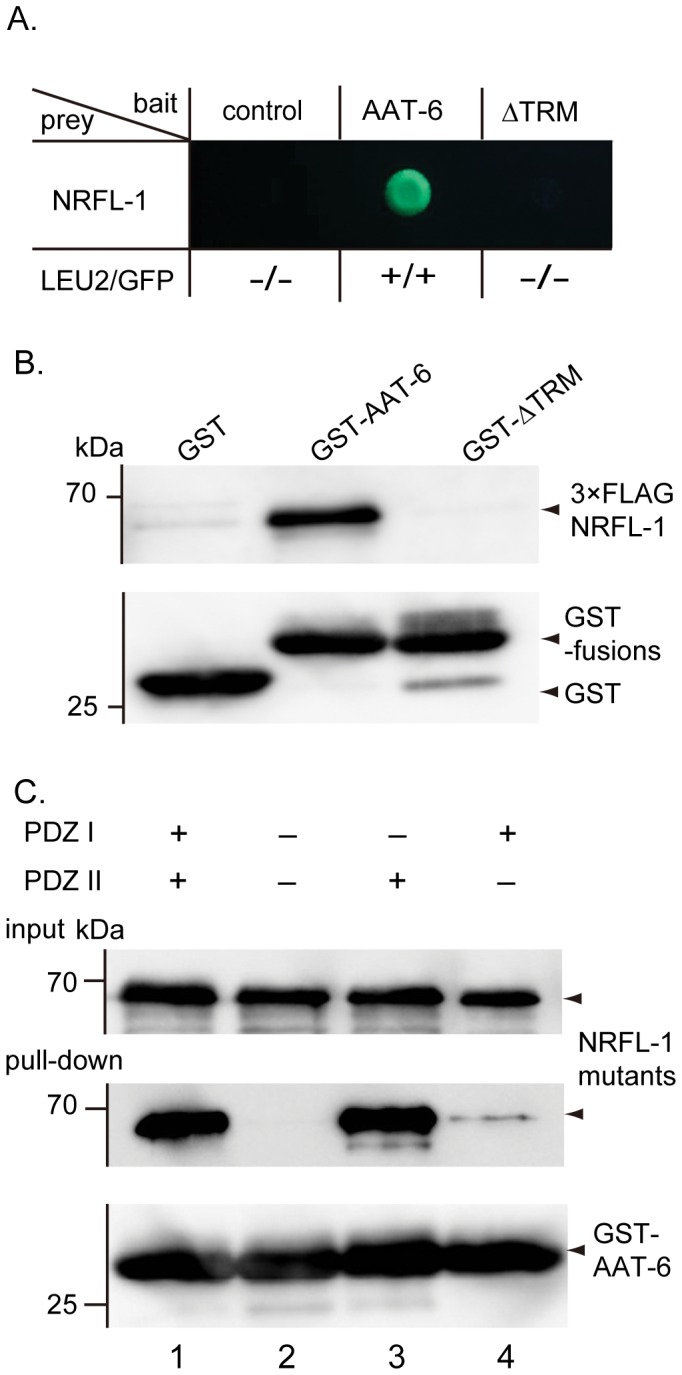
PDZ interaction between NRFL-1 and AAT-6. ***A***, Yeast-two hybrid assay. Full-length NRFL-1 was used as a prey, whereas the empty bait vector (*control*), AAT-6 *C*-terminus tail corresponding to residues 487–523 (*AAT-6*), and AAT-6 *C*-terminus tail without PDZ bindings motif (Δ*TRM*) were used as baits. Each bait-prey pair was assessed for LEU2 and GFP reporters. The pair of AAT-6 *C*-terminus tail and NRFL-1 grew on the medium without leucine and glowed. Similar results were obtained in three different experiments. ***B***, GST pull-down assay. 3×FLAG-NRFL-1 was pulled down by GST fusion of AAT-6 *C*-terminus tail corresponding to residues 487–523 (*GST-AAT-6*), but not by GST (*GST*) or GST fusion of AAT-6 *C*-terminus tail without PDZ binding motif (*GST-*Δ*TRM*). *Upper*, pull-down products probed by anti-FLAG antibody. *Lower*, pull-down products reprobed by anti-GST antibody. A representative blot from three experiments is shown. ***C***, Domain analysis by GST pull-down assay. Lanes 1, 2, 3 and 4 are for 3×FLAG-NRFL-1 (wild type), 3×FLAG-NRFL-1 with PDZ I/PDZ II both mutated (G26A/Y27A and E154A/F155A), 3×FLAG-NRFL-1 with PDZ I mutated (G26A/Y27A), and 3×FLAG-NRFL-1 with PDZ II mutated (E154A/F155A), respectively. GST fusion of AAT-6 *C*-terminus tail corresponding to residues 487–523 (*GST-AAT-6*) pulled down 3×FLAG-NRFL-1 (wild type) and 3×FLAG-NRFL-1 with PDZ II mutated but not 3×FLAG-NRFL-1 with PDZ I/PDZ II both mutated and 3×FLAG-NRFL-1 with PDZ II mutated, suggesting that PDZ II is the preferred domain for interacting with AAT-6. + and – in the figure denote wild type and mutated domain, respectively. *Upper*, input (1/10 of sample volume); *middle*, pull-down products probed by anti-FLAG antibody; *bottom*, pull-down product reprobed by anti-GST antibody. A representative blot from three experiments is shown.

To analyze further the interaction between AAT-6 and NRFL-1 found in the yeast two-hybrid matching, we first tested the involvement of AAT-6 *C*-terminal PDZ binding motif (-T-R-M) in the interaction. The yeast two-hybrid and the GST pull-down assay demonstrated that the deletion of the PDZ binding motif (-T-R-M) abolished the interaction of AAT-6 with NRFL-1 (Fig. 2A and B), showing its participation in the interaction. Next, to identify which PDZ domain of NRFL-1 is responsible for the interaction, we disrupted the PDZ domains of NRFL-1 by introducing mutations into the conserved carboxylate-binding loop: G26A/Y27A for PDZ I, E154A/F155A for PDZ II [27]. When both domains were mutated, the mutant was unable to bind with GST-AAT-6, further confirming the involvement of PDZ interaction (Fig. 2C). The mutation in PDZ I alone retained the binding, whereas the mutation in PDZ II alone greatly impaired the binding (Fig. 2C). Collectively, the *C*-terminus of AAT-6 preferentially binds to the PDZ II domain of NRFL-1.

### NRFL-1 is Expressed in the Intestine and Phosphorylated *in vivo*

**Figure 3:**
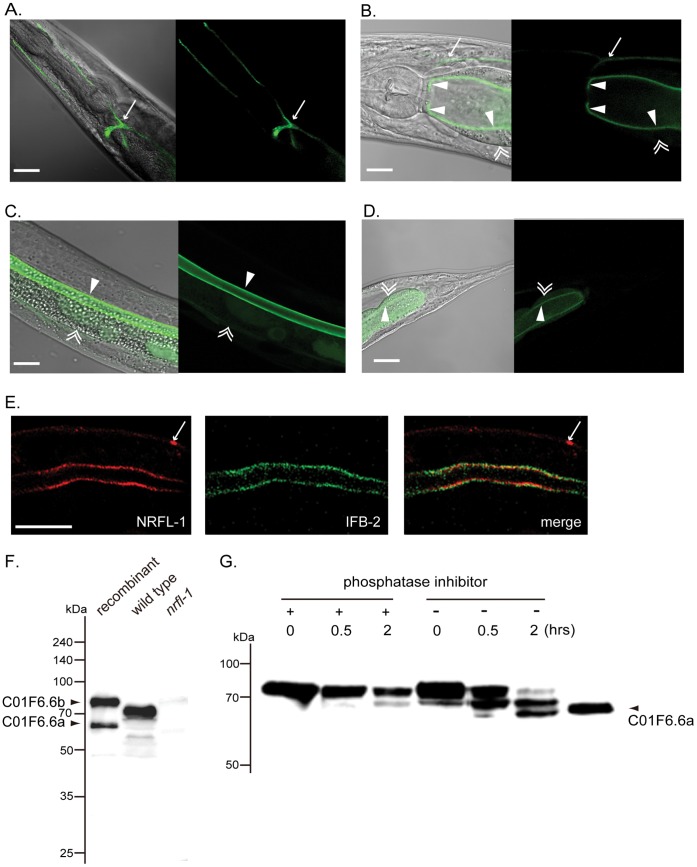
Expression and protein profile of NRFL-1 (C01F6.6) in *C. elegans*. *A* *–* ***D***, Expression of NRFL-1 in worm. *gfp::nrfl-1* expression was detected in excretory canal (*arrow* in ***A*** and ***B***) and luminal membrane of intestinal epithelial cells (*single-arrowhead* ) in the anterior (***B***), middle (***C***) and posterior (***D***) intestine. *gfp::nrfl-1* was not detected on the basal side (*double-arrowhead* in ***B***, ***C*** and ***D***). In ***A***, a worm with *gfp::nrfl-1* expression restricted to the excretory system was imaged for clarity. Such worms occasionally occurred in the transgenic population. In ***C*** and ***D***, cytosolic dispersion of NRFL-1 was seen. Scale bars, 25 µm. Ten worms examined for each. ***E***, Luminal enrichment of NRFL-1. Endogenous NRFL-1 was detected by anti-NRFL-1 antibody along the luminal side of the intestine. Non-specific signal on the body-wall is arrowed (*left*). IFB-2 was immune-labeled in a similar pattern (*middle*). Merged image shows that NRFL-1 is distributed apical to IFB-2 (*right*). Scale bar, 25 µm. Confocal images of a representative intestinal section (whole-worm) from seven independent experiments are shown. ***F***, Immunoblot of endogenous and recombinant NRFL-1. Left (*recombinant*), middle (*wild type*) and right (*nrfl-1*) lanes are for recombinant C01F6.6a and C01F6.6b proteins (untagged), lysate from wild-type, and lysate from *nrfl-1*(*tm3501*), respectively. Note that the band at ∼72 kDa in the middle lane was not detected in the right and the band did not match either C01F6.6a (∼62 kDa) or C01F6.6b (∼78 kDa). Sixty microgram of protein was loaded for the lysates. A representative blot from three separate experiments is shown. ***G***, Dephosphorylation of endogenous NRFL-1. The wild-type lysate was incubated with (+) and without (−) phosphatase inhibitors for the indicated period. Over incubation, the bands for inhibitor-free lysate migrated towards a position corresponding to the recombinant C01F6.6a. Ninety microgram of protein was loaded for the lysate. The results are confirmed in duplicate experiments.

A *gfp* reporter gene was fused to *nrfl-1* by employing the fosmid recombineering technology [28], which enables to incorporate larger genomic DNA into the construct and to cover as many *cis-*regulatory elements as necessary to reproduce accurate expression *in vivo*. *gfp*::*nrfl-1* was expressed in the excretory canal (Fig. 3A and B), the worm counterpart of the mammalian renal tubules, and the intestine (Fig. 3B, pharynx-anterior intestine; 3C, middle intestine; 3D, posterior intestine). Although previous large-scale gene expression profiling efforts using *nrfl-1-promoter::gfp* constructs observed GFP signals in the pharynx, intestine, excretory cells, and some tail cells [29], [30], our *gfp::nrfl-1* was exclusively expressed in the intestine and the excretory system. This pattern stayed consistent throughout the larval and adult stages. Using a specific antibody raised against the NRFL-1 (C01F6.6a) protein, endogenous NRFL-1 was detected in the luminal side of the intestine (Fig. 3E, and Fig. S1). However, the excretory canal was not stained with the antibody. NRFL-1 is found apical to IFB-2 (Intermediate Filament, B family-2) which localizes just beneath the intestinal microvilli [31], suggesting the enrichment in the intestinal microvilli (Fig. 3E).

Immunoblotting revealed a ∼72 kDa band which was not detected in the *nrfl-1* null lysate (Fig. 3F). *E. coli*-derived recombinant C01F6.6a protein and C01F6.6b protein were used as markers to determine which of the variants would correspond to the ∼72 kDa band (Fig. 3F). However, neither of the variant markers matched. This observation motivated us to consider the possibility that the NRFL-1 protein is post-translationally modified. To test the idea, we used phosphatase inhibitors to inhibit endogenous phosphatases and examined the expected band-shift due to dephosphorylation by incubating the fresh worm lysate at 37°C in the presence or absence of the phosphatase inhibitors. In the absence of the inhibitors, the bands gradually shifted to a position which corresponded to that of the recombinant C01F6.6a marker toward the end of inclubation, while the bands largely stayed at the original position in the presence of the inhibitors (Fig. 3G). This migration shift likely reflected progressive dephosphorylations of NRFL-1 and/or removal of modifications of NRFL-1 which is mediated by a separate protein in phosphorylation-dependent manner. The ∼72 kDa band, which was dominant *in vivo*, was, thus, due to posttranslational modifications of NRFL-1.

### Physical Interaction *in vivo*

**Figure 4:**
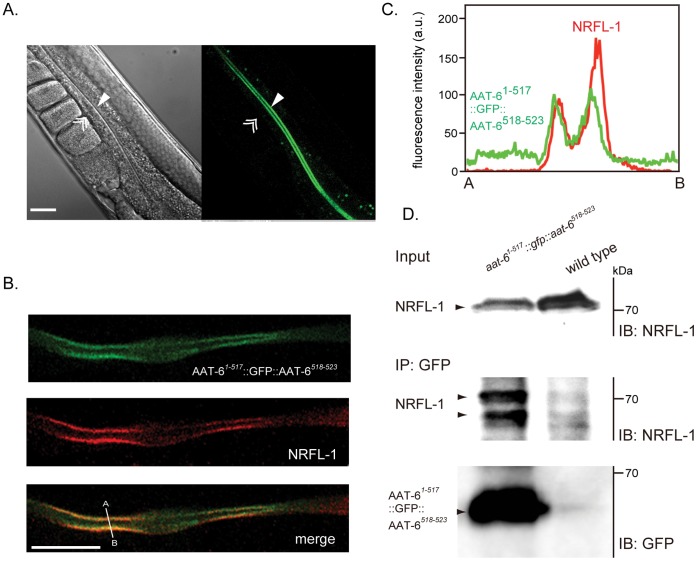
Interaction of AAT-6 with NRFL-1 *in vivo*. *A* , Expression of AAT-6 in worms. AAT-6*^1−517^*::GFP::AAT-6*^518–523^* was localized to the luminal surface (*single arrowhead*) but not to the basal side (*double arrowhead*) of the intestinal epithelia. Scale bar, 25 µm. Non-specific fluorescence on gut granules was seen. More than twenty worms were analyzed. ***B***, Co-localization of AAT-6 and NRFL-1. GFP fluorescence from AAT-6*^1−517^*::GFP::AAT-6*^518–523^* (*top*) was co-localized with immunostaining of NRFL-1 by anti-NRFL-1 antibody visualized by Cy3-labeled secondary antibody (*middle*). Bottom image is merged from top and middle images. Confocal images of a representative intestine section (whole worm) are shown. Scale bar: 25 µm. More than five worms were analyzed. ***C***, Intensity profile along the line A–B in the merged image shows an overlapping of the peaks of the NRFL-1and the AAT-6 signal. ***D***, Immunoprecipitation of NRFL-1/AAT-6 complex from worm lysate. The worm lysate was immunoprecipitated with anti-GFP monoclonal antibody (mouse) and immunoblotted using anti-NRFL-1 antibody. *Top*: input (2.5%). *Middle* and *bottom*: immunoprecipitant was immunoblotted using anti-NRFL-1 antibody and anti-GFP antibody (chicken), respectively. In the middle blot, two major bands of NRFL-1 detected (*arrowheads*), probably reflecting partial dephosphorylation during immunoprecipitation process. A representative blot of two separate experiments is shown.

To examine the interaction of AAT-6 and NRFL-1 *in vivo*, we first determined the precise subcellular localization of AAT-6 in the intestine. Because *gfp::aat-6* was not expressed and the product of *aat-6::gfp* was not localized on the plasma membrane, we instead inserted *gfp* into the region corresponding to the position between glutamine 517 and phenylalanine 518 in the *C*-terminal cytoplasmic tail of AAT-6 with the PDZ-binding motif intact. The reporter gene *aat-6^1−517^::gfp::aat -6^518–523^* was expressed and its product was localized to the luminal surface in the intestine (Fig. 4A). No other organs showed positive GFP signals. Immunofluorescence staining using anti-NRFL-1 antibody detected NRFL-1 on the intestinal luminal membrane (Fig. 3E). Combining the immunostaining of endogenous NRFL-1 and the confocal imaging of the AAT-6*^1−517^*::GFP::AAT-6*^518–523^* translational fusion, NFRL-1 and AAT-6 were detected with fluorescence overlapping, suggesting that they share their luminal localizations (Fig. 4B and C). To examine whether NRFL-1 and AAT-6 are in a protein complex *in vivo*, we immunoprecipitated AAT-6*^1−517^*::GFP::AAT-6*^518–523^* in the presence of phosphatase inhibitors using anti-GFP monoclonal antibody. We detected NRFL-1 in the sediment (Fig. 4D), confirming that NRFL-1 and AAT-6 are physically interactive *in vivo*.

### The Loss of NRFL-1 Deteriorates the Membrane Localization of AAT-6 Over Age

Studying consequences of the *nrfl-1* deletion to the NRFL-1/AAT-6 complex, we transferred an extrachromosomal array carrying *aat-6^1−517^*::*gfp*::*aat-6^518–523^* to *nrfl-1* mutants. We crossed *aat-6*(*tm2881*) carrying the extrachromosomal array against *nrfl-1*(*tm3501*);*aat-6*(*tm2881*), obtaining an extrachromosomal-array-carrying heterozygote: *nrfl-1*/+;*aat-6*. The heterozygote was selfed, yielding homozygous siblings carrying *aat-6^1−517^*::*gfp*::*aat-6^518–523^*. For *nrfl-1*(*ok2292*) carrying *aat-6^1−517^*::*gfp*::*aat-6^518–523^*, similarly, a sibling strain carrying both intact *nrfl-1* and the reporter gene was prepared as control. Siblings were used for analysis since they were thought to have minimal genetic differences. No noticeable differences in gross anatomy and growth within the sibling strains were observed (data not shown).

**Figure 5:**
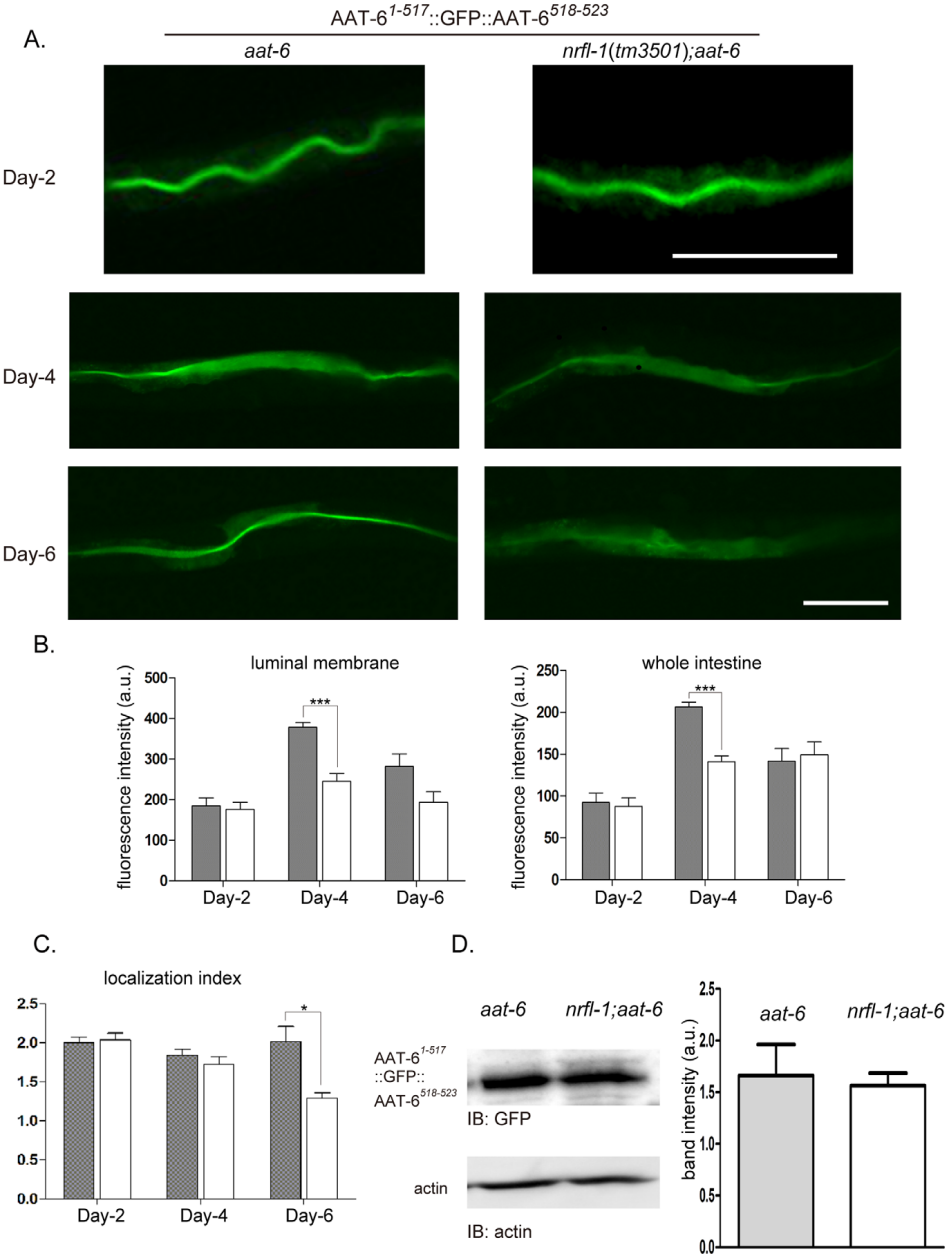
Maintenance of AAT-6 on the intestinal luminal membrane by NRFL-1. *A* , The localization of AAT-6*^1−517^*::GFP::AAT-6*^518–523^* was compared between *aat-6* and *nrfl-1*(*tm3501*);*aat-6* genetic backgrounds. Epifluorescence images of the distribution of AAT-6 in the intestine are shown for worms two, four and six days after hatching. In six-day old worm, the membranous localization of AAT-6 decayed in *nrfl-1*(*tm3501*);*aat-6*, whereas AAT-6 was retained on the luminal membrane in six days in the presence of NRFL-1 (*aat-6*). Scale bars: 100 µm. Representative pictures from more than ten worms analyzed for each are shown. ***B***, Fluorescence intensity was measured to quantify the age-dependent regulation. The intensity of the intestinal luminal surface was peaked at day four with significantly stronger signal in *aat-6* (gray column) compared with *nrfl-1*(*tm3501*);*aat-6* (white column). The intensity at day six did not differ significantly between the strains (*luminal membrane*). ***C***, The fluorescence intensity of the whole intestine showed a similar pattern (*whole intestine*). The localization index, luminal intensity divided by whole intestine intensity, quantifies the membranous localization. The six-day old *nrfl-1*(*tm3501*);*aat-6* worm had a significantly lower score, showing age-dependent decay in luminal localization (*localization index*). Gray column, *aat-6*. White column, *nrfl-1*(*tm3501*);*aat-6*. Values are presented with mean ± S.E. (n = 5). ***D***, Immunoblot and densitometric analysis of AAT-6*^1−517^*::GFP::AAT-6*^518–523^* in six-day old worm. Densitometric analysis followed by anti-GFP antibody immunobloting exhibited no significant difference in band intensity between the genetic backgrounds. A representative blot was presented with actin as a loading control. The bar graph indicates the relative band intensities of the respective sample. Values are presented with mean ± S.E. (n = 4).

The impact of the absence of NRFL-1 on the AAT-6 localization was assessed by epifluorescence imaging. In both *nrfl-1* and *nrfl-1*;*aat-6* genetic backgrounds, AAT-6 tagged with GFP (AAT-6*^1−517^*::GFP::AAT-6*^518–523^*) localized to the luminal surface of the intestinal tube till four-day old, at which the transgenic worms began to lay eggs (Fig. 5A). However, six-day old worms with *nrfl-1*;*aat-6* background failed to retain AAT-6 at the luminal membrane, whereas in *aat-6*, AAT-6 fluorescence remained along the luminal membrane (Fig. 5A). The luminal fluorescence intensity of AAT-6*^1−517^*::GFP::AAT-6*^518–523^* reached a peak at day 4 with significant difference between *aat-6* and *nrfl-1*;*aat-6*. The fluorescence intensity of whole intestine showed a similar pattern to the luminal intensity (Fig. 5B). The luminal intensity of *aat-6* at day 6 was not significantly higher than that of *nrfl-1*;*aat-6*. Localization index is defined as luminal membrane intensity divided by whole intestine intensity, quantifying the degree of membrane localization. High index values imply tight membranous localization whereas the value of 1 means complete diffusion. At day 6, AAT-6 was tightly localized to the luminal membrane in *aat-6* in contrast to the weak membranous localization in *nrfl-1*;*aat-6* (localization index: 2.08 vs. 1.29; *p*<0.05) (Fig. 5C). There were no significant differences in localization index at day 2 and 4. Consistent with the fluorescence intensities of the whole intestine at day 6, immunoblot analysis for six-day old worms showed no significant difference in the protein amount of AAT-6 between *aat-6* and *nrfl-1*;*aat-6* (Fig. 5D). Combined with the lower localization index, these data suggest that the intracellular fluorescence in the six-day old *nrfl-1*;*aat-6* worm is due to AAT-6 diffused in the cytoplasm in the absence of NRFL-1 (Fig. 5A).

A second mutant *nrfl-1*(*ok2292*) also demonstrated a similar decay in luminal membrane localization over age; the membranous localization of AAT-6 was maintained at day 2 and day 4 and deteriorated at day 6 (Fig. S2A). The luminal intensity was significantly lower in *nrfl-1*(*ok2292*) at day 6 as well as the whole intestine intensity (Fig. S2B). Compared with the pair of *aat-6* and *nrfl-1*(*tm3501*);*aat-6*, non specific gut-granules appeared particularly more evident along the basal intestinal membrane in this pair (Fig. S2A). Localization index was significantly lower in *nrfl-1*(*ok2292*) at day 4 and day 6 (Fig. S2C). The index for *nrfl-1*(*ok2292*) at day 6 was as low as 1.07, suggesting a nearly complete diffusion.

### The Loss of NRFL-1 Accelerates the Decay of Membranous Localization of AAT-6 Over Age

Worms were followed up to ten-day old. The membranous localization of AAT-6 completely decayed by day 10 both in *aat-6* and *nrfl-1*(*tm3501*);*aat-6* (Fig. S3A). In the other experimental pair (*nrfl-1*(*ok2292*) and its control), similarly, the localization became blurry in *control* strain (Fig. S3B). However, about 20% of the control strain still retained AAT-6 on the membrane (Fig. S3B, *bottom*). Regardless of the presence of NRFL-1, AAT-6 appeared to fail to localize to the membrane in very advanced age. Such decay in the luminal localization is accelerated in mutants lacking *nrfl-1* (Fig. 5, S2 and S3).

### NRFL-1 Limits the Mobility of AAT-6 on the Membrane

**Figure 6:**
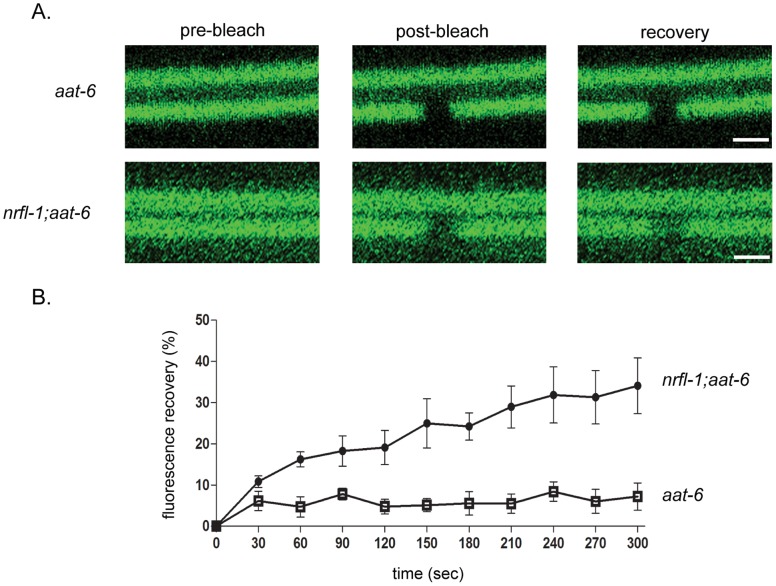
Immobilization of AAT-6 on the intestinal apical membrane by NRFL-1. ***A***, FRAP analysis of the AAT-6 dynamics was performed in 4-day old worms. Confocal images of AAT-6 (AAT-6*^1−517^*::GFP::AAT-6*^518–523^*) was compared between *aat-6* and *nrfl-1*;*aat-6* genetic backgrounds. Top pictures indicate representative images prior to photobleaching (*pre-bleach*), immediately after photobleaching (*post-bleach*), and 300 sec after photobleaching (*recovery*). ***B***, Graph depicts the time course of recovery of AAT-6 fluorescence for *nrfl-1*;*aat-6* (•) and *aat-6* (□). Recovery was measured with the pre-bleach fluorescence intensity being 100% and the post-bleach intensity being 0%. The recovery curves were generated from 5 separate experiments and the values were expressed as mean ± S.E. (n = 5). Scale bars: 2 µm.

The dynamic status of AAT-6 in the membrane might be dependent on NRFL-1. To examine the effect of NRFL-1 on the mobility of AAT-6 in the membrane, we carried out a fluorescence recovery after photobleaching (FRAP) analysis in four-day old worms in which AAT-6 is still on the membrane with or without NRFL-1. AAT-6 was mostly immobile when NRFL-1 was present (Fig. 6A and B). In the absence of NRFL-1, a FRAP experiment revealed an exchange of the AAT-6 molecules within the intestinal luminal membrane, ∼30% recovery of fluorescence intensity in 300 seconds (Fig. 6B). The increased fluorescence recovery is consistent with the static maintenance of AAT-6 in the luminal membrane of the intestine by NRFL-1.

## Discussion

In splice variants of NRFL-1, the double-domain isoform was identified as the dominant *nrfl-1* gene product in this study (Fig. 3F and G). We found that AAT-6, a member of the *C. elegans* AAT (amino acid transporter) family (an orthologue of the mammalian SLC7 family), binds to the PDZ II domain of NRFL-1. In mammals, it has been shown that the PDZ binding motif -D-S/T-X-L has particularly high affinity to the PDZ I domains of NHERF1 and NHERF2 [32]. In contrast, the PDZ binding motif (-C-T-R-M) of AAT-6 preferentially binds to the PDZ II domain of NRFL-1 which is the less homologous to the PDZ domains of mammalian NHERF1 and NHERF2 (Fig. 1A and Table S1). It is interesting that the PDZ II domain of NRFL-1 may be more adapted to ligands relatively uncommon for mammalian NHERFs such as amino acid transporters.

Immunoblot analysis revealed that NRFL-1 is multiply-phosphorylated (Fig. 3G), similar to NHERF1 which is constitutively phosphorylated [33]. Phosphorylation of NHERF1 has various consequences in its function and localization. Phosphorylated NHERF1 is more localized in the cytoplasm rather than to the plasma membrane and has weaker affinity to ligands such as CFTR, platelet-derived growth factor (PDGF) receptor, and β_2_-adrenergic receptor [34]. Phosphorylation of NHERF1 regulates the intramolecular interaction in which the *C*-terminus of NHERF1, which also constitutes a PDZ-binding motif, binds to the second PDZ domain [35]. While the significance of phosphorylation in NRFL-1 is left to future studies, it is possible that phosphorylation and dephosphorylation of NRFL-1 have some functional relevance through the regulation of protein-protein interactions and macromolecule complex building.

The advantage of *C. elegans* NRFL-1 in the study of PDZ scaffold is that NRFL-1 is the single worm orthologue of NHERF family, so that the studies in *C. elegans* should be less susceptible to the redundancy problem which we encounter in mammals. In *Drosophila melanogaster*, Sip1 is the sole fly orthologue of NHERF protein [4], [36]. Sip1 has a single PDZ domain with 54% identity and 66% similarity to PDZ I of NRFL-1, and 48% identity and 61% similarity to PDZ II of NRFL-1. In fly embryo, Sip1 is found along the luminal side of the epithelium in the intestine, wing imaginal disc, and follicle cells [36]. Despite the similar protein profile of Sip1 with NRFL-1, the *Sip1*-null fly fails to hatch to larva or die shortly after advancing to the larval stage with remarkably impaired epithelium integrity [36], [37]. Sip1 regulates development of the embryonic epithelium by interacting with actin, moesin, and sterile-20 kinase Slik [36]. In contrast, the *nrfl-1* mutants are viable with normal gross appearance, consistent with findings from genome wide RNAi screenings in which the interferences of *nrfl-1* do not elicit morphological or lethal phenotypes [38]–[40]. The viability of the *nrfl-1* mutants is an obvious advantage because we are able to study a NHERF-related protein throughout development in a system with low genetic redundancy.

In this study we found that NRFL-1 regulates AAT-6 in two aspects: maintenance of its membrane localization (Fig. 5 and S2) and immobilization on the luminal membrane (Fig. 6). Until early breeding stage (up to four-day old), AAT-6 stays on the membrane with or without NRFL-1 (Fig. 5 and S2), and NRFL-1 immobilizes AAT-6 on the membrane (Fig. 6). In late breeding stage (six-day old), in contrast, NRFL-1 is crucial in retaining AAT-6 at the apical membrane; the absence of NRFL-1 causes the internalization of AAT-6. In further advanced age (ten-day old), the deterioration of the membranous localization of AAT-6 occurs in the majority of worms carrying NRFL-1 (Fig. S3), suggesting that such deterioration is a natural course. Localization index, which measures how tightly AAT-6 is membrane-localized, declines with age in the *nrfl-1*mutants; it is not the case with the control worms (Fig. 5C and S2C). This suggests that a loss of AAT-6 membranous localization is accelerated with age and that NRFL-1 protects AAT-6 from such aging process. When the interaction with NHERF proteins is disrupted, some membrane proteins are susceptible to internalization and degradation [41]–[43]. As for NRFL-1/AAT-6 complex, the protein amount of AAT-6 remains unchanged in *nrfl-1*;*aat-6* worm even at six days old, suggesting that the loss of NRFL-1 does not cause the degradation of AAT-6 (Fig. 5D). Without NRFL-1, AAT-6 somehow stays intracellularly without being degraded. While the mechanism underlying age-dependent maintenance of AAT-6 on the intestinal luminal membrane has yet to be clarified, NRFL-1 with two PDZ domains may further bind unidentified proteins essential to keep AAT-6 in the intestinal apical membrane in aged phase.

Taking advantage of the amenability of live-animal imaging in *C*. *elegans*, a FRAP analysis was applied to the NRFL-1/AAT-6 complex *in vivo* to further examined the role of NRFL-1 in the maintenance of AAT-6 in the membrane (Fig. 6). To our knowledge, this is the first report to apply FRAP analysis *in vivo* to the study of PDZ interactions involving NHERF proteins [21], [44]. In the presence of NRFL-1, the fluorescence recovery of AAT-6 reaches to plateau in 30 s, leaving ∼95% of immobilized fraction. The absence of NRFL-1 results in an increased fluorescence recovery of ∼35% in 5 min, suggesting that AAT-6 is immobilized by NRFL-1 (Fig. 6). For human parathyroid hormone receptor PTH1R, it was proposed that the receptor in the plasma membrane is immobilized by NHERF1 anchored to the actin cytoskelton through ERM (Ezrin-Radixin-Moesin) proteins [44]. The distribution of the worm orthologue ERM-1 completely overlaps with NRFL-1: the excretory canal and the luminal surface of the intestine [45], where ERM-1 participates in brush border formation [46]. The physical association between NRFL-1 and ERM-1 in a yeast two-hybrid experiment has been reported [47]. Recently, it was revealed that ERM proteins recognize the *C*-terminal 13 residues of NHERF1 or 2 for interaction and that the binding sequence is characterized as Motif-1: MDWXXXXX(L/I)FXX(L/F), where X denotes any amino acid [48]. NRFL-1 has a *C*-terminal 13-residue similar to Motif-1: MSLNEKYQLVSNM, underpinning the yeast two-hybrid finding. Therefore, it is reasonable to assume that NRFL-1 immobilizes AAT-6 through the interaction with ERM proteins such as ERM-1.

The NHERF-interacting CFTR, whose *D*-value (diffusion coefficient) was estimated to be 0.99±0.09×10^−10^ cm^2^/s, has a half time recovery of 1–1.2 min for a bleached circle of ∼5 µm diameter in MDCK cells [22]. In contrast, AAT-6 does not reach a 50% recovery in 5 min of observation for ∼2 µm bleached circle. AAT-6 shows a much slower recovery in worm than CFTR does in MDCK cells. Our experiment was performed at 25°C, a physiological temperature for *C*. *elegans*, whereas the CFTR diffusion was measured at 37°C. The temperature is a considerable determinant of molecular motility since the viscosity heavily depends on the temperature. A temperature hike from room temperature (∼25°C) to 37°C observes two to four-fold increases in the diffusion coefficients of plasma membrane proteins such as epidermal growth factor receptor in MDCK cells [49]. Differences in the apical surface architecture between MDCK cell and worm intestinal epithelium should also be taken into account. Estimating from cross-section images, worms have 4–6 microvilli in a 1-µm segment of the intestinal brush border [50], whereas in MDCK cells there are as few as 1 or 2 microvilli in 1 µm [51]. The abundance of microvilli in worm is likely a topological constraint hindering molecular diffusions along the membrane. Otherwise, though it is not mutually exclusive, yet unidentified molecules which limit the motility may be involved in this phenomenon.

In summary, we found that NRFL-1, the sole *C*. *elegans* orthologue of mammalian NHERF family, is crucial in protecting AAT-6 from the decrement in the intestinal luminal membrane associated with aging as well as in immobilizing the transporter on the membrane. *C*. *elegans*, which is transparent and has a fast life cycle, facilitated observations of the age-associated change in localization of a transporter supported by scaffold protein. Since NRFL-1 has two PDZ domains, it likely has more binding partners. There might be interactors which interplay with NRFL-1 in an age-dependent manner.

## Materials and Methods

### Strains and Maintenance

Strains were seeded with *E.coli* OP50 on NGM agar plates and maintained under standard conditions at 20°C [52]. For large-scale culture, worms were grown in liquid culture as described [53] and were isolated by sucrose flotation [54].

Alleles used were N2 Bristol wild type, *nrfl-1*(*tm3501*), *nrfl-1*(*ok2292*), *aat-6*(*tm2881*), and *nrfl-1*(*tm3501*);*aat-6*(*tm2881*). After isolated from the TMP/UV library [55], *nrfl-1*(*tm3501*) and *aat-6*(*tm2881*) were outcrossed eight and five times to N2, respectively. The resultant strains were crossed to generate *nrfl-1*(*tm3501*);*aat-6*(*tm2881*). N2 and *nrfl-1*(*ok2292*) was purchased from the Caenorhabditis Genetics Center. The *ok2292* strain was outcrossed three times against N2. The genotypes were confirmed by single worm PCR [56].

Transgenic lines generated were N2 *Ex*[*gfp::nrfl-1*, *rol-6*(*su1006*)], N2 *Ex*[*aat-6^1−517^::gfp::aat-6^518–523^*, *rol-6*(*su1006*)], *nrfl-1*(*tm3501*) *Ex*[*gfp::nrfl-1*, *rol-6*(*su1006*)], *nrfl-1*(*ok2292*) *Ex*[*aat-6^1−517^::gfp::aat-6^518–523^*, *rol-6*(*su1006*)], *aat-6*(*tm2881*) *Ex*[*aat-6^1−517^::gfp::aat-6^518–523^*, *rol-6*(*su1006*)], and *nrfl-1*(*tm3501*);*aat-6*(*tm2881*) *Ex*[*aat-6^1−517^::gfp::aat-6^518–523^*, *rol-6*(*su1006*)].

### Yeast Two-hybrid Constructs and Two-hybrid Matching

The *C*-terminal end of AAT-6 corresponding to residues 487–523 was obtained by PCR using pcDNA3.1-AAT-6 (full-length) as a template. The fragment was inserted into EcoRI and XhoI sites of the bait vector pEG202 (MoBiTec). For the AAT-6 bait with PDZ-binding motif deletion (ΔTRM), the cDNA fragment of AAT-6 corresponding to residues 487–520 was obtained by PCR and inserted to pEG202.

The fragments of C01F6.6a (full-length), C09H6.2a (residues 14–954), C25F6.2a (full-length), C27A2.6 (residues 9–518), C35D10.2 (residues 1–327), F26D11.11a (residues 113–679), F30F8.3 (full-length), F44D12.1 (residues 4–1094), F44D12.4 (full-length), T14G10.2a (residues 419–1311), and T21G5.4 (full-length) were obtained by PCR using the cDNA clones yk1663b04, yk669c4, yk591g3, yk606b11, yk720d3, yk524b7, yk1680d03, yk324a9, yk712g5, yk45b10, and yk438c5 as templates, respectively. The templates were a generous gift of Dr. Yuji Kohara (National Institute of Genetics, Mishima, Japan). The template for C35D10.2, yk720d3, yielded a point-mutated PCR product with A to C alteration at 464 bp, which results in amino acid substitution N155T. The mutation was corrected using the standard PCR-based mutagenesis technique. The fragments of C01F6.6b (full-length), C25F6.2b (full-length), C25G4.6 (amino acids: 1–271), F30A10.8b (full-length), and T26E3.3a (full-length) were obtained by PCR using a *C. elegans* cDNA library (*C. elegans* whole adult cDNA library, MoBiTec) as a template. F44D12.1, T14G10.2a, and T26E3.3a were cloned into EcoRI and XhoI sites of the bait vector pEG202 by using In-Fusion cloning system (Clontech). Other fragments were inserted into EcoRI and XhoI sites of pJG4-5 prey vector (MoBiTec).

The Lex-A-based GFP yeast-two hybrid system (Glow’n’Grow system, MoBiTec) was employed to match AAT-6 and one of the sixteen candidate PDZ proteins. Transformation and assay procedure followed manufacturer’s instructions. The EGY48 yeast strain was transformed with pEG202-AAT-6 (487–523), pGNG I (a GFP reporter vector, MoBiTec), and pJG4-5 carrying one of the prey proteins. The transformed colonies were first assayed for growth on medium lacking leucine. Growing colonies were further assayed for GFP expression in a dark room under a handy UV lamp (wave length: 365 nm, Model UVGL-25, UVP Inc.).

### Expression and Purification of Recombinant Proteins

The cDNA fragment corresponding to residues 487–523 of AAT-6 was cloned between BamHI and XhoI sites of pGEX-6P-1 (GE Healthcare). The plasmid was transformed into *E. coli* strain BL21 (DE3). The transformed bacteria were grown in LB medium at 37°C until *A*_600_ between 0.4 and 0.6. Protein expression was induced by 0.1 mM of isopropyl β-*D*-thiogalactopyranoside for 90 minutes. The bacteria were lysed by sonication in 20 mM Tris-HCl pH 8.0, 100 mM NaCl, 1 mM EDTA and Complete Protease Inhibitor Cocktail (Roche). The lysate was centrifuged at 5,000×*g* for 15 minutes to remove debris. The supernatant was further ultracentrifuged at 353,000×*g* for 15 minutes. The supernatant was applied to a chromatography column (Poly-Prep Chromatography Columns, BioRad) packed with Glutathione Sepharose 4B beads (GE Healthcare). Washing and elution procedure followed the manufacturer’s instructions. The eluate was dialyzed against 50 mM Tris-HCl pH 7.4 with 0.1 mM DTT. The final product (GST-AAT-6), supplemented with glycerol (final concentration 10% (v/v)), was stored at −80°C until use. Preparation of AAT-6 with PDZ-binding motif deletion (GST-ΔTRM) also followed the same protocol.

The construct for NRFL-1 was prepared in two steps. The cDNA for full-length wild type NRFL-1 was first ligated into p3×FLAG-myc-CMV-24 (Sigma-Aldrich) as a HindIII-NotI fragment. The fragment including 3×FLAG coding region and NRFL-1 cDNA was next ligated into pGEX-6P-1 as a BamHI-XhoI fragment. The construct was transformed into *E. coli* BL21 to express the recombinant protein GST-3×FLAG-NRFL-1. Only a small fraction of the recombinant protein (∼5%) appeared soluble. The supernatant after ultracentrifuge, which contained the soluble fraction of the protein, was GST-affinity purified as described above. For GST-tag removal, the GST fusion protein was treated with PreScisson protease (GE Healthcare) in 20 mM Tris-HCl pH 7.0, 150 mM NaCl, 1 mM EDTA, 1 mM dithiothreitol, and 0.01% NP-40 at 4°C overnight. The resulting 3×FLAG-NRFL-1 protein remained soluble and was stored at −80°C until use. For the constructs of NRFL-1 mutants (G26A/Y27A, E154A/F155A, and G26A/Y27A/E154A/F155A), the conventional PCR-based mutagenesis technique was applied using pGEX-6P-1 3×FLAG-NRFL-1 as a template. Expression, purification, and GST-tag removal followed the same protocol for the wild type.

Protein concentrations were estimated by BCA Protein Assay Kit (Thermo Scientific).

### Antibody Generation

The full-length NRFL-1 (C01F6.6a) was cloned at SmaI and XhoI sites of pET-47b(+) (Novagen). The recombinant protein, expressed as described above, was collected as pellet of inclusion body. The pellet was resolved in 4 M urea in PBS (137 mM NaCl, 2.7 mM KCl, 4.3 mM Na_2_HPO_4_, 1.47 mM KH_2_PO_4,_ pH 7.4). The solution was dialyzed against PBS and the resolved protein precipitated. The sediment was resolved in 2 M urea in PBS and centrifuged at 15,000×*g* for ten minutes. The supernatant was used as an antigen. A female New Zealand white rabbit was immunized with 200 µg of antigen for the first shot. Three boost shots with 100 µg of antigen each were followed at a two-week interval. The animal was sacrificed for total blood collection. The serum was affinity purified by using an antigen-coupled column (HiTrap NHS-activated HP column, GE Healthcare); the final product contained 0.26 mg/ml of IgG (by UV absorbance at 280 nm).

### GST Pull-down Assay

For GST pull-down assay, 2 µg of GST fusion protein and 5 µg of recombinant NRFL-1 wild-type or mutant protein were incubated overnight at 4°C in 50 mM Tris pH 7.4, 150 mM NaCl, 5 mM EDTA, and 0.1% NP-40. After washing the sediment five times with the incubation buffer, the sample was separated on a 10% polyacrylamide gel, immunoblotted, and probed with anti-FLAG rabbit polyclonal antibody (Sigma; dilution 1∶20,000). The membrane was reprobed with anti-GST mouse monoclonal antibody (Santa Cruz; dilution 1∶10,000).

### Transgenic Lines

Transformation of worms followed the standard protocol of exogenous DNA microinjection into the gonads [57].

The λ-Red-based fosmid recombineering technique was used to make transforming constructs [28]. The recombineering kit was kindly gifted by Dr. Oliver Hobert (Columbia University). In brief, for the *nrfl-1* reporter gene, the fosmid WRM063dG02 was electroporated into *E. coli* strain SW105 [58] (distributed by Biological Resource Branch, NCI). A DNA segment containing *gfp*, *FRT/Flp* sites, and *galK* selectable marker was recombinated at the immediate upstream of the start codon by heat shock-inducible λ Red recombinase. Arabinose inducible Flp recombinase removed the *galK* marker as well as a part of the *FRT/Flp*, resulting in a translational fusion GFP::NRFL-1. A similar strategy to generate an *N*-terminal GFP fusion of AAT-6 was attempted. However, no expression was observed. Instead, we inserted *gfp* into the region corresponding to the cytosolic tail with the *C*-terminal PDZ-binding motif intact. A GFP tag flanking bi-directional four-glycine linkers (4×glycine-GFP-4×glycine) was inserted between glutamine 517 and phenylalanine 518 using a *gfp* cassette against the fosmid WRM0631dC09, resulting in AAT-6(1–517)-4×G::GFP::4×G-FKCTRM (denoted by AAT-6*^1−517^*::GFP::AAT-6*^518–523^*).

Five ng/µl of the recombineered fosmids were co-injected with 50 ng/µl of pRF4 and worm genomic DNA (PvuII digested). The microinjection solution contained 150 ng/µl of DNA.

For each construct, multiple transmissible lines were established and one representative line was used for the analysis. *nrfl-1*(*tm3501*);*aat-6*(*tm2881*) *Ex*[*aat-6^1−517^*::*gfp*::*aat-6^518–523^*, *rol-6*(*su1006*)] was generated by crossing *aat-6*(*tm2881*) *Ex*[*aat-6^1−517^*::*gfp*::*aat-6^518–523^s*, *rol-6*(*su1006*)] to *nrfl-1*(*tm3501*);*aat-6*(*tm2881*). A *nrfl-1*(*ok2292*) *Ex*[*aat-6^1−517^*::*gfp*::*aat-6^518–523^*, *rol-6*(*su1006*)] was outcrossed against N2, yielding *nrfl-1*(*ok2292*) *Ex*[*aat-6^1−517^*::*gfp*::*aat-6^518–523^*, *rol-6*(*su1006*)] and a sibling strain which carries the intact *nrfl-1* and the *gfp* reporter genes.

### Protein Extraction and Dephosphorylation-inhibition Assay

Protein extraction was performed following Gendrel et al. [59] with minor modification. Around 70 µl of frozen worm pellet was ground in a mortar and pestle immersed in liquid nitrogen and thawed in 300 µl of ice-cold homogenization buffer (50 mM HEPES pH 7.6, 50 mM KCl, 2 mM MgCl_2_, 250 mM sucrose, 1 mM EDTA, and 6 µl of Complete Protease Inhibitor Cocktail (Roche) solution (two tablets in 0.5 ml of H_2_O)). After brief sonication, the sample was cleared at 20,000×*g* for 15 min. The supernatant was equally split into an experimental group and a control group. The experimental group was supplemented with 20 µl of PhosSTOP phosphatase inhibitor cocktail (Roche) solution (two tablets in 1 ml of H_2_O), while the control group was left untreated. The samples were incubated at 37°C for 0, 30, or 120 min. Upon completion of incubation, the sample was boiled at 95°C for 5 min with Laemmli buffer [60]. The sample was separated on a 10% polyacrylamide gel, blotted, and detected by using anti-NRFL-1 antibody (dilution 1∶500).

### Co-immunoprecipitation

Immunoprecipitation using anti-GFP antibody was performed following Gendrel et al. [59]. Around 300 µl of frozen worm pellet was ground as described above, thawed in 500 µl of the homogenization buffer, briefly sonicated, and centrifuged at 5,000×*g* for 15 min at 4°C. For input sample, 20 µl of the lysate was kept aside and frozen at −80°C during the immunoprecipitation procedure. The supernatant was brought up to 2 ml with the homogenization buffer supplemented with 0.5% Triton X-100, 100 mM NaCl, and 30 µl of PhosSTOP phosphatase inhibitor cocktail (Roche) solution. After rotating for 15 min at 4°C, the sample was centrifuged at 12,000×*g* for 20 min to remove insoluble materials. Five microgram of anti-GFP mouse monoclonal antibody (Wako) was added to the supernatant. After overnight incubation at 4°C, 70 µl of Protein G slurry (Sigma-Aldrich) was added. After 90 minutes incubation at 4°C, the sample was washed twice with 25 mM HEPES pH 7.6, 100 mM NaCl, and 1 mM EDTA and subjected to immunoblotting for NRFL-1 detection as described above. AAT-6*^1−517^*::GFP::AAT-6*^518–523^* was probed with anti-GFP chicken antibody (Aves Labs. dilution 1∶10,000).

### Immunoblot and Densitometric Analysis

At four-day old, worms carrying *Ex*[*aat-6^1−517^*::*gfp*::*aat-6^518–523^*, *rol-6*(*su1006*)] in the *nrfl-1*(*tm3501*);*aat-6*(*tm2881*) and the *aat-6*(*tm2881*) backgrounds were picked up under a fluorescence microscope and further cultured for another two days. For each preparation, 150 six-day-old worms were used. After washed with PBS and frozen in liquid nitrogen, the frozen worm pellet was thawed in PBS and briefly sonicated. The sample was incubated, with occasional agitation, at 37°C for 30 min with Laemmli buffer. Immunoblotting with anti-GFP antibody was performed as described above. For loading control, actin was probed with anti-β-Actin mouse antibody (Sigma-Aldrich. dilution 1∶10,000). The band intensity was quantified by Multi Gauge software (Fujifilm). Data was processed by a statistical software Prism (GraphPad software). The data are expressed as the means ± S.E. (n = 4).

### Immunofluorescence Labeling

For pre-absorption, *nrfl-1*(*tm3501*) was fixed in a 1∶1 (v/v) mixture of 2% (w/v) paraformaldehyed in PBS and methanol for 90 min at 4°C and briefly sonicated. Anti-NRFL-1 antibody was prepared in PBST (PBS supplemented with 1% bovine serum albumin, 0.5% Triton X-100, and 1 mM EDTA) at a dilution of 1∶25. Anti-NRFL-1 antibody in PBST was incubated with the fixed *nrfl-1*(*tm3501*) worm overnight at 4°C. After centrifuge, the supernatant was used as the primary antibody.

To study the subcellular localization of NRFL-1 relative to IFB-2, anti-IFB-2 monoclonal antibody (MH33) [61] was used at 1∶50. For co-localization study, *aat-6* (*tm2881*) *Ex*[*aat-6^1−517^*::*gfp*::*aat-6^518–523^*, *rol-6*(*su1006*)] worms were fixed as described above. The fixed worms were processed as described elsewhere [62]. The worms were, then, treated overnight with the primary antibody followed by Cy3-labeled donkey anti-rabbit IgG (H+L) (Jackson ImmunoResearch Laboratories) as a secondary antibody (dilution: 1∶200) at 4°C. The sample was prepared for observation as described [63].

### Confocal Microscopic Observation of Worms

Observations were made using a LSM 510 Meta laser scanning confocal microscope with a Plan-Apochromat 63×/1.4 oil immersion objective lens (Zeiss). For expression pattern analysis, worms anesthetized in 0.4% NaN_3_ in M9 were examined under a 488 nm laser line for GFP excitation. Co-localization imaging was carried out under a 488 nm laser line for GFP and a 543 nm laser line for Cy3, respectively. Intensity profile was analyzed by ImageJ (National Institutes of Health, Bethesda, Maryland, USA, http://imagej.nih.gov/ij/, 1997–2012).

### Epifluorescence Imaging

Two-day, four-day, six-day, and ten-day old worms were pick up from a synchronized colony of *aat-6*(*tm2881*) *Ex*[*aat-6^1−517^*::*gfp*::*aat-6^518–523^*, *rol-6*(*su1006*)], *nrfl-1*(*tm3501*);*aat-6*(*tm2881*) *Ex*[*aat-6^1−517^*::*gfp*::*aat-6^518–523^*, *rol-6*(*su1006*)], *nrfl-1*(*ok2292*) *Ex*[*aat-6^1−517^*::*gfp*::*aat-6^518–523^*, *rol-6*(*su1006*)], or the sibling strain of *nrfl-1*(*ok2292*) *Ex*[*aat-6^1−517^*::*gfp*::*aat-6^518–523^*, *rol-6*(*su1006*)]. Epifluorescence imaging was carried out by an Olympus BX61 microscope equipped with the MetaMorph version 6.1 software (Molecular Devices) [64]. Worms were anesthetized as above for imaging. All the worms were imaged under identical imaging parameters: UplanSApo 10× objective; exposure time 30 msec for *tm3501* and 70 msec for *ok2292*; 1×1 binning.

For quantification of the fluorescence intensity, ImageJ was used for demarcation of the region of interest and measurement of the intensity and the area. The intestine and the luminal surface were demarcated. The intensity and the area of the demarcated regions were measured. After subtracted by the background intensity, the mean intensity (the raw intensity divided by the area) was calculated for each sample. The localization index was defined as the mean luminal surface intensity divided by the mean intestine intensity. Data was processed by Prism. The data are expressed as the means ± S.E.

### Fluorescence Recovery after Photobleaching (FRAP) Analysis

To prepare worms with minimal movement during observation, four-day old worms bearing up to five eggs were anesthetized by immersing in 0.4% NaN_3_ in M9 for 30 min [65]. FRAP experiments were carried out in a room air-conditioned at 25°C, using a LSM 510 Meta laser scanning confocal microscope with a Plan-Apochromat 63×/1.4 oil immersion objective lens. An intestinal epithelium cells around the vulva were subjected to photobleach invasion. A circular spot of ∼2 µm on the luminal surface of the intestine was photobleached by 20 iterations of the 488 nm laser with 100% laser power transmission. The subsequent time-lapse images were acquired at 0.05% transmission (a 30-second interval up to 300 seconds). Images were collected at a 12-bit intensity resolution over 512×512 pixels at a pixel dwell time of 3.2 µsec. For each strain, five immobilized worms were studied. All the images were acquired under the identical microscope and camera setting. The fluorescence recovery of the region of interest was calculated as [*f*(*t*)-*f*_post_]/[*f*_pre_-*f*_post_], where *f*_pre_ denotes the fluorescence intensity before photobleaching, *f*_post_ denotes the fluorescence intensity immediately after photobleaching, and *f*(*t*) denotes the fluorescence intensity at *t* seconds after photobleaching [66]. Data was processed by Prism. The data are expressed as the means ± S.E.

## Supporting Information

Figure S1 **Specific immunostaining of NRFL-1 in** ***C. elegans*** **.** Anti-NRFL-1 antibody stained the luminal membrane of intestinal tubes (*arrow heads*) of wild type worms, whereas it failed to stain the intestine of *nrfl-1*(*tm3501*) with occasional non-specific stains in ruptured body wall (*arrows*). The staining along the body wall in the wild type worm in the right panel is considered non-specific.
(TIF)

Figure S2 **Recapitulation of the decay in the apical localization of AAT-6 in a second mutant** ***nrfl-1*** **(** ***ok2292*** **).** ***A***, As observed in *nrfl-1*(*tm3501*);*aat-6,* the membranous localization of AAT-6 became blurry in *nrfl-1*(*ok2292*). The control strain is a sibling of the experimental *nrfl-1*(*ok2292*) strain which carries intact *nrfl-1.* Compared with *nrfl-1*(*tm3501*);*aat-6*, gut granules were more evident particularly in old worms in *nrfl-1*(*ok2292*) and its control. Scale bars: 100 µm. Representative pictures from more than ten worms analyzed for each are shown. ***B***, Significantly stronger fluorescence was observed on the intestinal luminal surface at day six in the control (gray column) compared with *nrfl-1*(*ok2292*) (white column) (*luminal membrane*). The intestinal fluorescence was also stronger at day six (*whole intestine*). ***C***, The localization indexes, luminal intensity divided by intestinal intensity, were higher at day four and six, recapitulating a similar pattern observed in *nrfl-1*(*tm3501*);*aat-6* (*localization index*). Gray column, *control*. White column, *nrfl-1*(*ok2292*). Values are presented with mean ± S.E. (n = 5).
(TIF)

Figure S3 **The membrane retention of AAT-6 in ten-day old worm.** ***A***, The distribution of AAT-6 was followed up to day ten to determine whether the loss is a normal occurrence. In the *aat-6* worm, the membranous localization of AAT-6 was completely lost by day ten. Scale bars: 100 µm. Representative pictures from more than ten worms analyzed for each are shown. ***B***, In *nrfl-1*(*ok2292*) and its control, typically, AAT-6 disappeared from the membrane (*top*). In ∼20% of the 10-day old control worm, the membrane retention was still preserved (*arrowed, bottom*). Scale bars: 100 µm. Representative pictures from more than nine worms analyzed for each are shown.
(TIF)

Table S1 **Comparison between NRFL-1 and, PDZK1 and IKEPP.** Two PDZ domains of NRFL-1 was compared with PDZ domains of human PDZK1 (519 amino acids) and human IKEPP (505 amino acids). PDZK1 and IKEE have four PDZ domains in tandem. For each domain comparison, identity/similarity (%/%) was assigned as described in Figure 1. BLAST searches using PDZK1 and IKEPP as query also converged to NRFL-1. However, the identity/similarity values are lower than those assigned to NHERF1 and 2 (Fig. 1C).
(DOC)
