# Supplementary figures and images for: NRFL-1, the C. elegans NHERF Orthologue, Interacts with Amino Acid Transporter 6 (AAT-6) for Age-Dependent Maintenance of AAT-6 on the Membrane
Source: PLoS One. 2012 Aug 15;7(8):e43050. doi: 10.1371/journal.pone.0043050 (PMC3419730; doi:10.1371/journal.pone.0043050)

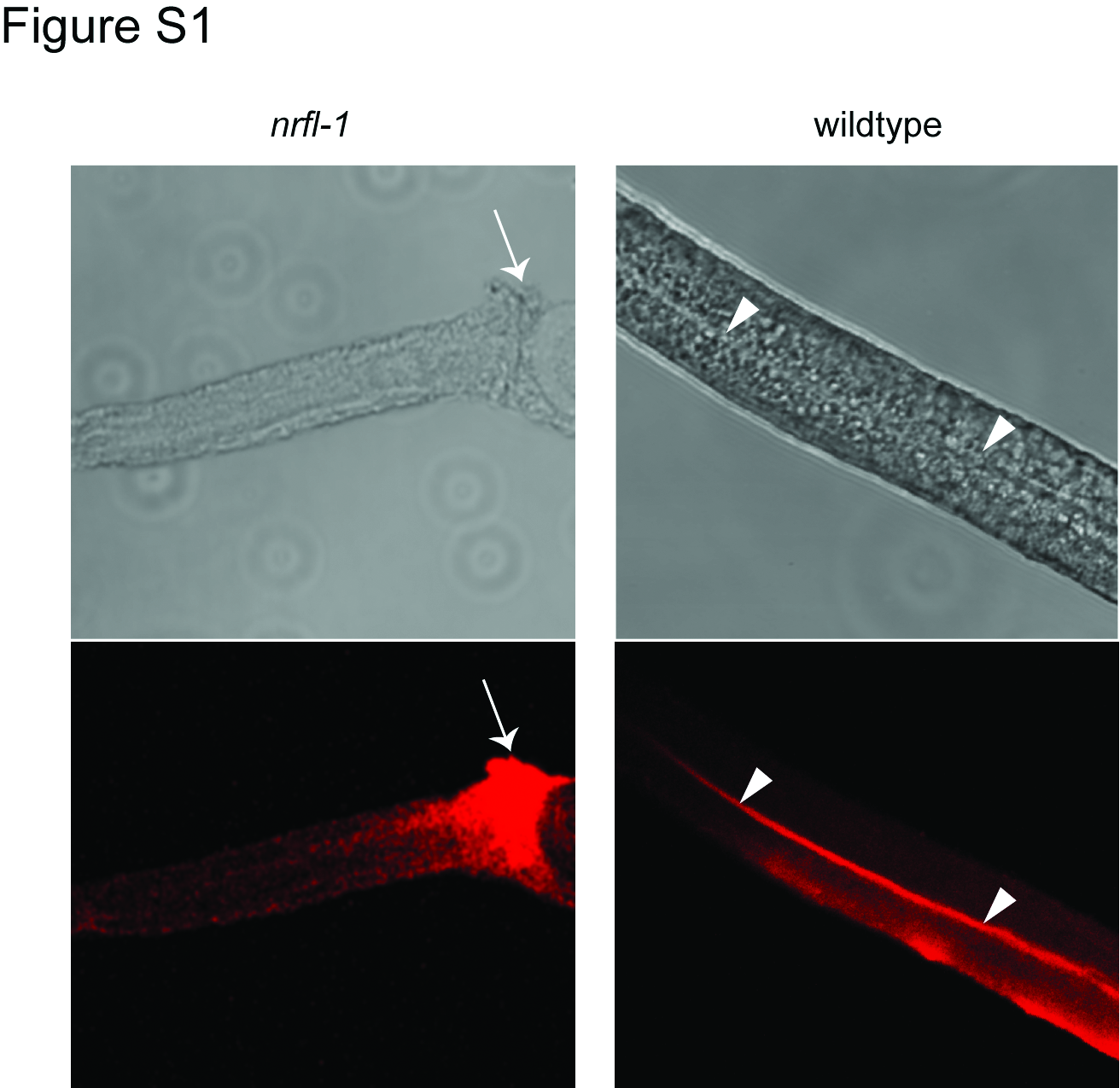

Supplement: Figure S1 — Specific immunostaining of NRFL-1 in C. elegans . Anti-NRFL-1 antibody stained the luminal membrane of intestinal tubes (arrow heads) of wild type worms, whereas it failed to stain the intestine of nrfl-1(tm3501) with occasional non-specific stains in ruptured body wall (arrows). The staining along the body wall in the wild type worm in the right panel is considered non-specific. (TIF) [file pone.0043050.s001.tif]

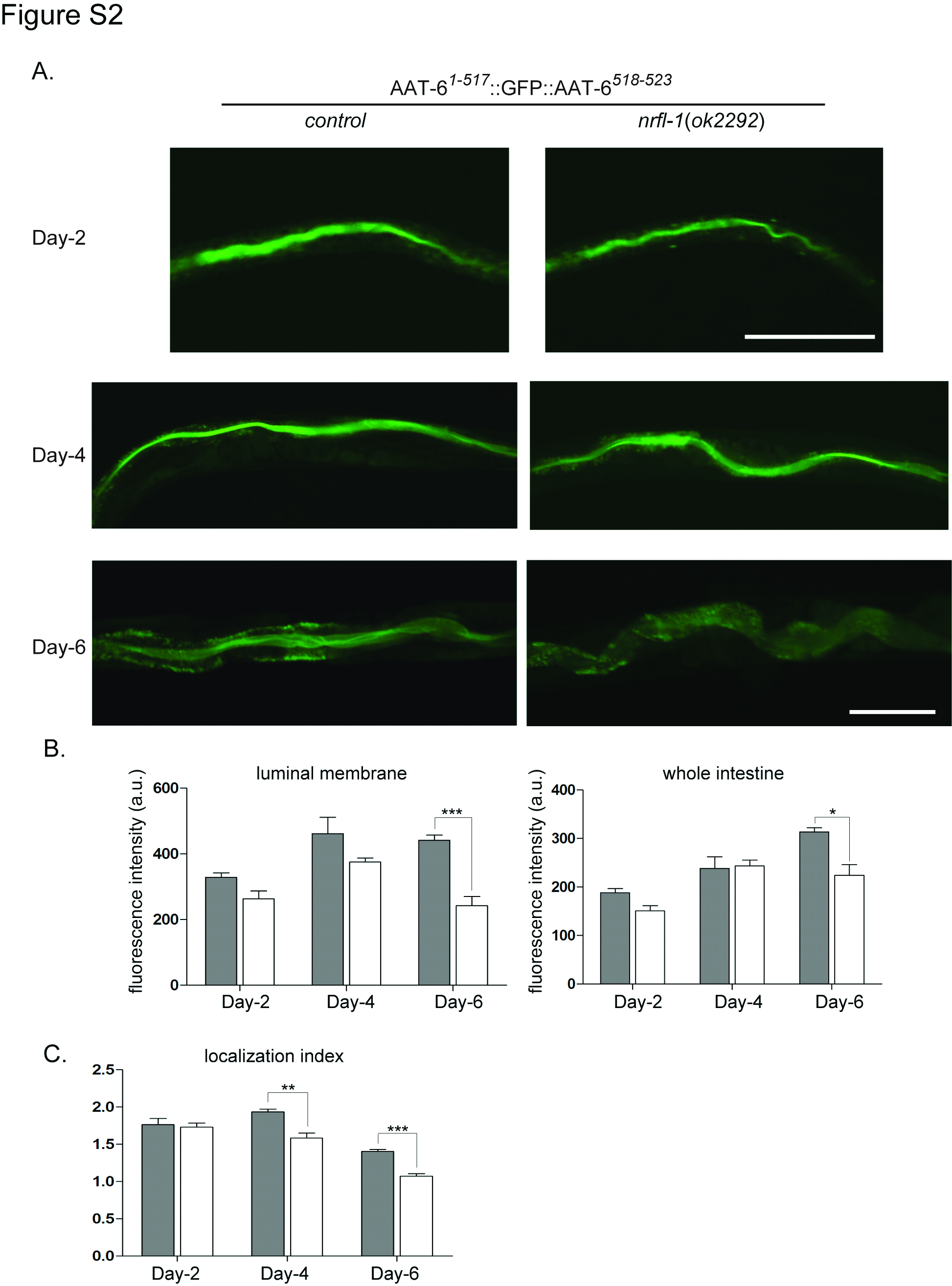

Supplement: Figure S2 — Recapitulation of the decay in the apical localization of AAT-6 in a second mutant nrfl-1 ( ok2292 ). A, As observed in nrfl-1(tm3501);aat-6, the membranous localization of AAT-6 became blurry in nrfl-1(ok2292). The control strain is a sibling of the experimental nrfl-1(ok2292) strain which carries intact nrfl-1. Compared with nrfl-1(tm3501);aat-6, gut granules were more evident particularly in old worms in nrfl-1(ok2292) and its control. Scale bars: 100 µm. Representative pictures from more than ten worms analyzed for each are shown. B, Significantly stronger fluorescence was observed on the intestinal luminal surface at day six in the control (gray column) compared with nrfl-1(ok2292) (white column) (luminal membrane). The intestinal fluorescence was also stronger at day six (whole intestine). C, The localization indexes, luminal intensity divided by intestinal intensity, were higher at day four and six, recapitulating a similar pattern observed in nrfl-1(tm3501);aat-6 (localization index). Gray column, control. White column, nrfl-1(ok2292). Values are presented with mean ± S.E. (n = 5). (TIF) [file pone.0043050.s002.tif]

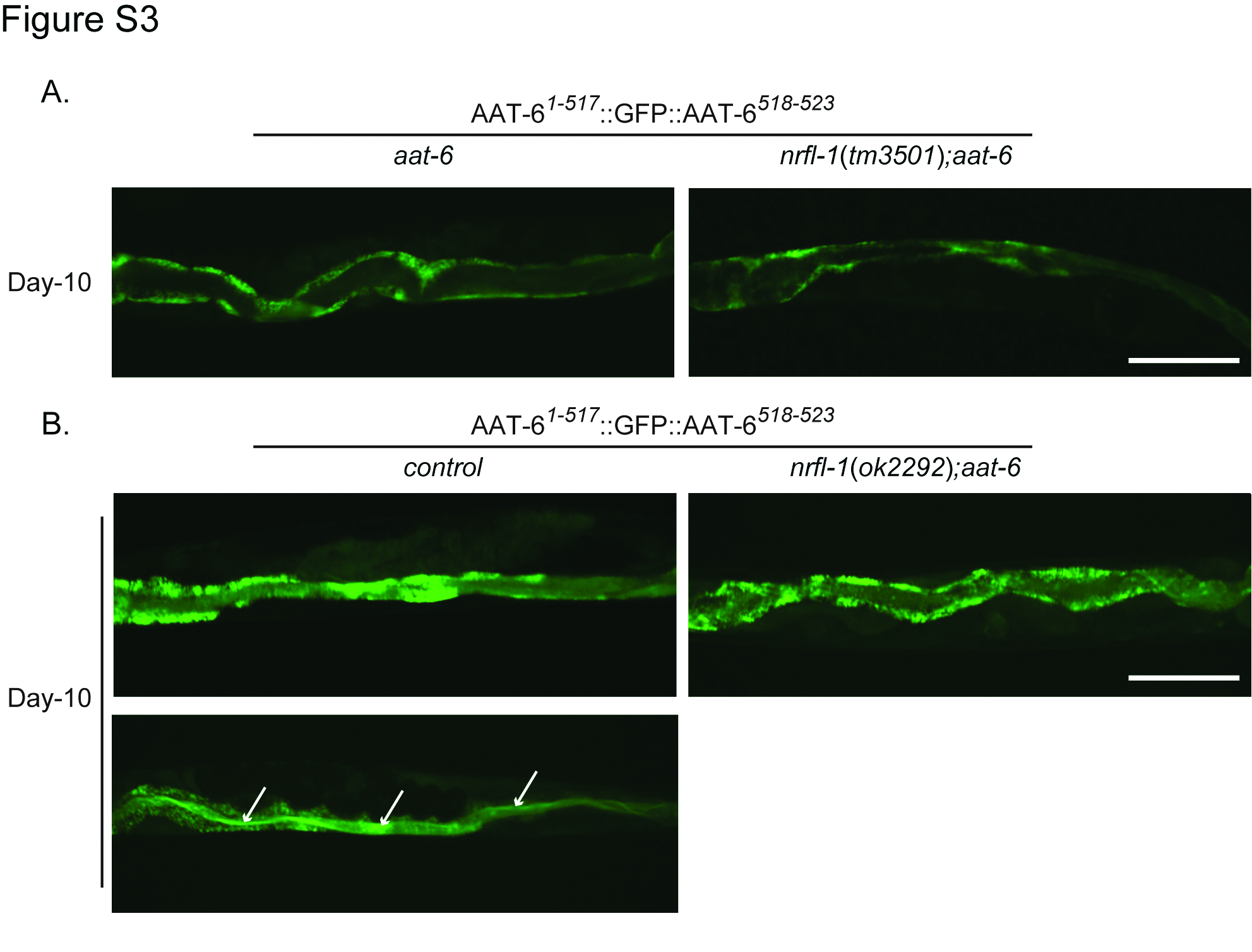

Supplement: Figure S3 — The membrane retention of AAT-6 in ten-day old worm. A, The distribution of AAT-6 was followed up to day ten to determine whether the loss is a normal occurrence. In the aat-6 worm, the membranous localization of AAT-6 was completely lost by day ten. Scale bars: 100 µm. Representative pictures from more than ten worms analyzed for each are shown. B, In nrfl-1(ok2292) and its control, typically, AAT-6 disappeared from the membrane (top). In ∼20% of the 10-day old control worm, the membrane retention was still preserved (arrowed, bottom). Scale bars: 100 µm. Representative pictures from more than nine worms analyzed for each are shown. (TIF) [file pone.0043050.s003.tif]
