# Supplementary material for: NRFL-1, the C. elegans NHERF Orthologue, Interacts with Amino Acid Transporter 6 (AAT-6) for Age-Dependent Maintenance of AAT-6 on the Membrane
Source: PLoS One. 2012 Aug 15;7(8):e43050. doi: 10.1371/journal.pone.0043050 (PMC3419730; doi:10.1371/journal.pone.0043050)
Supplement: Table S1 — Comparison between NRFL-1 and, PDZK1 and IKEPP. Two PDZ domains of NRFL-1 was compared with PDZ domains of human PDZK1 (519 amino acids) and human IKEPP (505 amino acids). PDZK1 and IKEE have four PDZ domains in tandem. For each domain comparison, identity/similarity (%/%) was assigned as described in Figure 1. BLAST searches using PDZK1 and IKEPP as query also converged to NRFL-1. However, the identity/similarity values are lower than those assigned to NHERF1 and 2 (Fig. 1C). (DOC) [file pone.0043050.s004.doc]

Table S1. Comparison between NRFL-1 and, PDZK1 and IKEPP.

|  | PDZK1 I | PDZK1 II | PDZK1 III | PDZK1 IV |
| --- | --- | --- | --- | --- |
| NRFL-1 I | 37/56 | 30/58 | 38/56 | 39/60 |
| NRFL-1 II | 36/52 | 26/55 | 34/55 | 38/51 |

|  | IKEPP I | IKEPP II | IKEPP III | IKEPP IV |
| --- | --- | --- | --- | --- |
| NRFL-1 I | 39/60 | 25/45 | 36/54 | 33/49 |
| NRFL-1 II | 34/52 | 30/52 | 36/52 | 31/44 |
